# Spread of networked populations is determined by the interplay between dispersal behavior and habitat configuration

**DOI:** 10.1073/pnas.2201553120

**Published:** 2023-03-09

**Authors:** Bronwyn Rayfield, Celina B. Baines, Luis J. Gilarranz, Andrew Gonzalez

**Affiliations:** ^a^Department of Biology, McGill University, Montreal, QC H3A 1B1, Canada; ^b^Department of Aquatic Ecology, Eawag (Swiss Federal Institute of Aquatic Science and Technology) 8600 Dübendorf, ZH, Switzerland

**Keywords:** spatial habitat configuration, habitat fragmentation, *Folsomia candida*, dispersal kernel, algebraic connectivity

## Abstract

Predicting the spread of populations across fragmented habitats is vital if we are to manage their persistence in the long term. We applied network theory with a model and an experiment to show that spread rate is jointly defined by the configuration of habitat networks (i.e., the arrangement and length of connections between habitat fragments) and the movement behavior of individuals. We found that population spread rate in the model was well predicted by algebraic connectivity of the habitat network. A multigeneration experiment with the microarthropod *Folsomia candida* validated this model prediction. The realized habitat connectivity and spread rate were determined by the interaction between dispersal behavior and habitat configuration, such that the network configurations that facilitated the fastest spread changed depending on the shape of the species’ dispersal kernel. Predicting the spread rate of populations in fragmented landscapes requires combining knowledge of species-specific dispersal kernels and the spatial configuration of habitat networks. This information can be used to design landscapes to manage the spread and persistence of species in fragmented habitats.

## Significance

Predicting how populations persist and spread in patchy and fragmented habitats is a research challenge with significant implications for the conservation and management of species. We combined theory and an experiment with the springtail *Folsomia candida* to show that population spread—the time taken for a population to colonize a network of habitat fragments—is predicted by the distances among habitat fragments and how easy it is for organisms to move between fragments. Depending on the pattern of connections among habitats and how frequently individuals successfully use them to move between habitat fragments, landscapes will promote spread in some species while impeding spread in other species. This knowledge can be used to manage real-world populations (e.g., reintroductions) in fragmented habitats.

In the context of biological invasions (1), extinction due to habitat loss (2), emerging diseases (3), and climate-driven range shifts (4), we have an urgent need to understand and predict the spread of populations. The discovery of factors that predict movement and population spread can be used to design landscapes that maintain native species (5, 6) and restrict the spread of invasive species and diseases (e.g., ref. 7). The erection of barriers to slow invasions (8, 9) or the addition of corridors to support dispersal and migration (10–12) are examples of control through landscape design. The success of these interventions requires models and indicators that link information about species-specific dispersal behavior and habitat spatial structure to predict spread rates and distribution.

Spatial networks (13–16) provide a powerful representation of patchy and fragmented habitats, where habitat patches (habitable nodes) are connected by movement paths and corridors (nonhabitable links). Studies have shown that habitat network configuration—the arrangement of habitat nodes and the links connecting them in patchy landscapes—can strongly determine population persistence (17–20), range size (21), abundance (22–24), and ecosystem functioning (25). Habitat network configuration has also been shown to influence the dynamics of population spread (26, 27). However, spatial population processes may not be simple reflections of habitat configuration; we hypothesized that knowledge of how the dispersal behavior of species (i.e., the probability of movement) interacts with the habitat network would be required to understand the dynamics of population spread. Conservation efforts rest on the need to understand how habitat networks and species movement ecology can be combined to predict spatial processes, and whether the same rules apply to different species.

Several pieces of information are needed to understand species’ dispersal behavior and spread on a network of habitats (28). Aspects of dispersal ability such as movement capacity and the ability to withstand inhospitable conditions (e.g., low food availability, suboptimal physical conditions, greater predation risk) dictate how dispersal success declines with distance and the link lengths that can be successfully traversed by a species (29, 30). Aspects of dispersal propensity also strongly influence the shape of the dispersal kernel (31). Some organisms move faster through inhospitable environments (e.g., ref. 32) to reduce the time spent at risk and/or because they spend time evaluating hospitable habitat patches for settlement. Species also vary in how willing they are to disperse (even if they have the capacity), how much they assess link properties such as distance before embarking on dispersal, and how likely they are to alter their behavior (e.g., reverse their path) once they have begun the dispersal process (29). All these factors contribute to the shape of a species’ dispersal kernel, which quantifies dispersal probability as a function of distance, and which varies across species. We expect that the shape of a species’ dispersal kernel will interact with habitat network configuration to determine the rate of spread across a network.

At the same time, network-based measures provide powerful metrics to quantify the connectivity of habitat networks. These connectivity metrics can be used to predict metapopulation persistence (20, 33) as well as rates of colonization (28, 34, 35). The connectivity (or adjacency) matrix D whose elements $d_{i,j}$ denote the strength of the link connecting habitat nodes i and j , is a fundamental mathematical representation of the metapopulation network (36, 37). The eigenvalues of the Laplacian matrix derived from the adjacency matrix D (38) are used to describe features related to global and mesoscopic network structure and dynamic interactions among network components. Here, we focus on algebraic connectivity, the second smallest eigenvalue of the Laplacian matrix (38). Algebraic connectivity is known to play an important role in many relevant dynamical processes on networks such as synchronization, diffusion, and extinction (39, 40) and was therefore a strong candidate for a metric to predict how quickly individuals move through a habitat network.

We combined theory and an experiment to study the spatial population dynamics of nonequilibrium populations expanding into spatial habitat networks with different link configurations. Ecological dynamics within and across nodes (e.g., population growth and spread) are known to be mediated by the degree of randomness of the links allowing dispersal between nodes of the spatial graph (41). By combining knowledge of dispersal behavior and habitat network configuration, we hypothesized that habitat network configuration influences the rate of spread, and this will be strongly explained by differences in the algebraic connectivity of networks.

We used a metapopulation model parameterized using experimental data describing population growth of the microarthropod *Folsomia candida* (42) that has been used as a biological model for movement and growth on networks (43). We then used the model to understand the movement and spread process on habitat networks of different configurations. We assessed whether spread in the model was predicted by habitat configuration and algebraic connectivity of the entire network. Algebraic connectivity predicted spread dynamics in both the model and experimental networks. We used the model to show that spatial spread dynamics observed during the experiment are well explained by linking the shape of the kernel describing the probability of movement between nodes to the spatial configuration of the habitat network.

## Modeling and Measuring the Effect of Habitat Configuration on Population Size and Spread

**Fig. 1.**
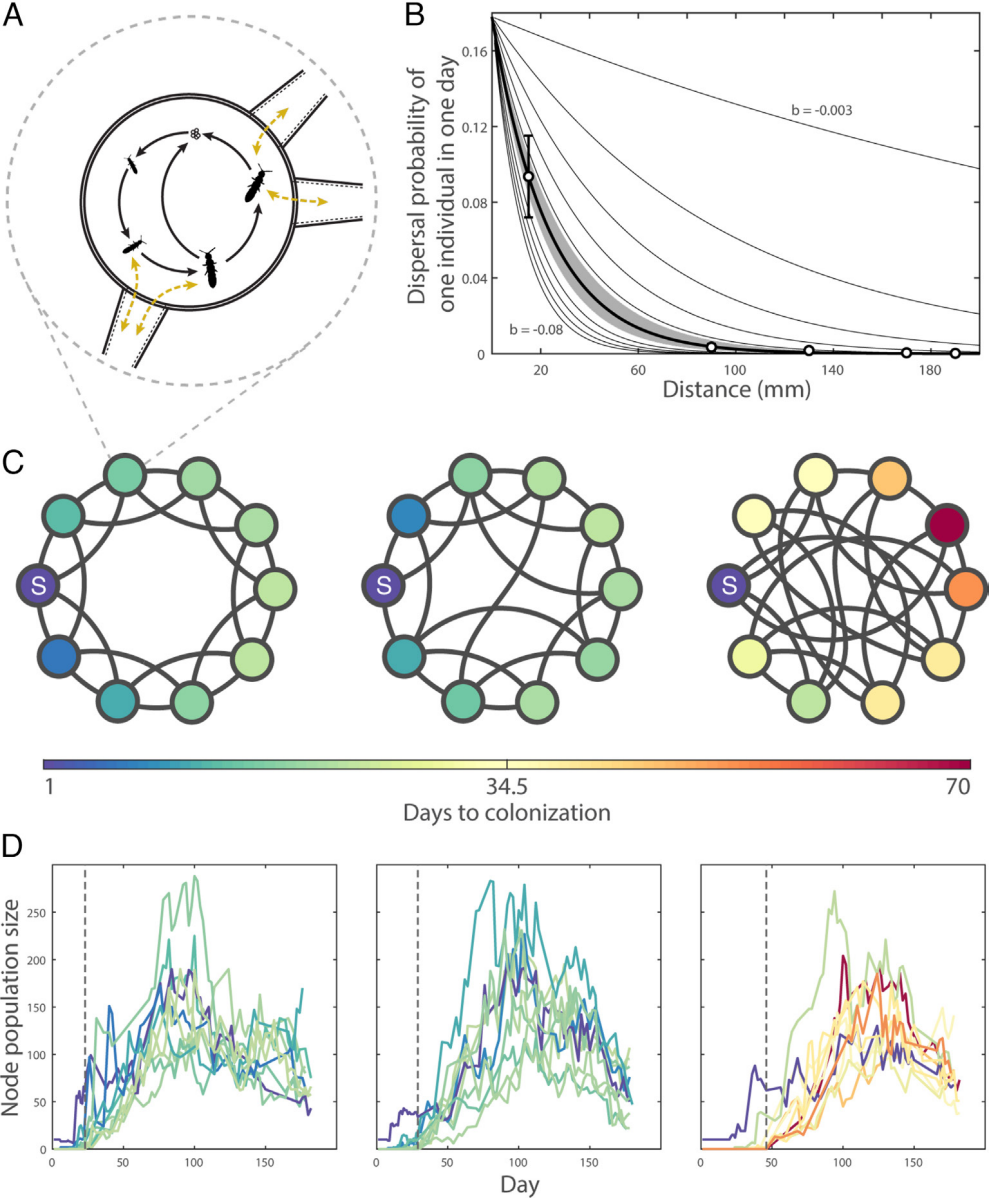
Habitat network configurations and demographic characteristics of the model system. (*A*) Depiction of *Folsomia candida*’s life cycle, size stages, reproduction, and dispersal. (*B*) Dispersal kernel. Open black circles show dispersal probabilities of *F. candida* estimated experimentally. The thick black line shows the negative exponential function fitted to these experimental data points; the grey shaded area shows the 95% CI of this fitted function. Thin black lines show alternative dispersal kernels used in the model. (*C* and *D*) Representative replicates of each of the three habitat configurations used in this study. From left to right: lattice, partially rewired, and random. (*C*) Network structure. Circles are nodes (habitat patches), and black lines are links (movement corridors). The diameter of nodes and lengths of links are shown to scale. “*S”* indicates the location of the source node—the initial location of the individuals added to the network at the start of the experiment. (*D*) Node population size over the course of the experiment; each line represents a single node. Vertical dashed lines indicate the number of days to full network occupancy. Color of circles (*C*) and lines (*D*) indicates the number of days it took for that node to be colonized.

To investigate the effects of habitat configuration on population size and spread, we designed habitat networks with three different levels of link rewiring [Fig. 1 and *SI Appendix*, Fig. S1, (44)]. In lattice configurations, all nodes were linked to their four nearest neighbors. Then, we created another set of networks by randomly rewiring 20% of the links while fixing node coordinates. We call these “partially rewired” networks. Finally, fully “random” networks were created by randomly rewiring all links of the lattice networks. All networks had the same number of nodes, links, and the same average degree. We used these three habitat configurations in both the population model and the laboratory experiment we describe below.

We created a stochastic population model parameterized for the life-history of the microarthropod *Folsomia candida* [a standard model organism (45)] that we used in the experiment [*Methods;* (42)]. We modelled the metapopulation dynamics taking place in the networks described above. To inoculate the landscapes, we introduced individuals into a single source node in each network (node *S* in Fig. 1*C*). Dispersal distances in *F. candida* are described by a negative exponential probability distribution with exponent *b* (*b* = −0.043 ± 0.007; thick black line + grey area in Fig. 1*B*; see *SI Appendix* for methods on estimating the dispersal kernel for *F. candida*). We developed a distance-dependent model in which dispersal probability between nodes was dependent on link length (i.e., a dispersal kernel), given by the dispersal kernel that was estimated for *F. candida*. The model captures essential information about individual movement encoded in the dispersal kernel and uses this to successfully predict the dynamics observed in our experiment. We compared the distance-dependent model to a distance-independent model in which we set the probability of dispersal to be constant across all links. To enable comparison between the two model versions, the dispersal probability of every link in the distance-independent model is equal to the average dispersal probability given the dispersal kernel with exponent b (*SI Appendix*, Methods).

We tested the predictions of our model by conducting a multigeneration experiment with *F. candida*, using physical habitat networks of the same configurations as those in the model. Link lengths in the experiment matched those used in the model. In the experiment, patches of artificial habitat were connected via flexible tubes providing nonhabitable corridors to form networks of nodes and links (*SI Appendix*, Fig. S1). *F. candida* individuals were then introduced—as in the model—into a single source node (node *S* in Fig. 1*C*) in each network at the start of the experiment. Food (in the form of granulated dry baker’s yeast) was applied to the nodes as a two-state (“food added” or “no food”) Markov series. The food added to each node therefore followed a unique feeding regime [*sensu* (46)]. This created spatiotemporally variable resource heterogeneity to encourage movement among the nodes (*SI Appendix*). We used automated image recognition analysis to count the number of individuals present in each node at a frequency of 1 to 2 (maximum 5) d over 182 d.

In both the model and experiment, we measured population size at the node level (the count of juvenile and adult springtails observed in a node; *SI Appendix*, Fig. S8) and the network level (the sum of the observed population sizes of all nodes comprising the network). At the network level, spread was defined as the time to full network occupancy (number of days from initiation of experiment to population size of all nodes in a network being greater or equal to 1; *SI Appendix*, Fig. S9).

Once the model was validated, we used it to determine the joint effects of habitat configuration and the shape of the dispersal kernel (described by the exponent *b*) on spread rate. We conducted simulations with varying values of *b* and predicted the rate of population spread on networks with different degrees of rewiring. Values of *b* close to zero indicate populations in which long-distance dispersal is common, while as *b* becomes more negative, long-distance dispersal becomes less likely (Fig. 1*B*).

## Habitat Network Configuration Predicts Time to Full Network Occupancy

**Fig. 2.**
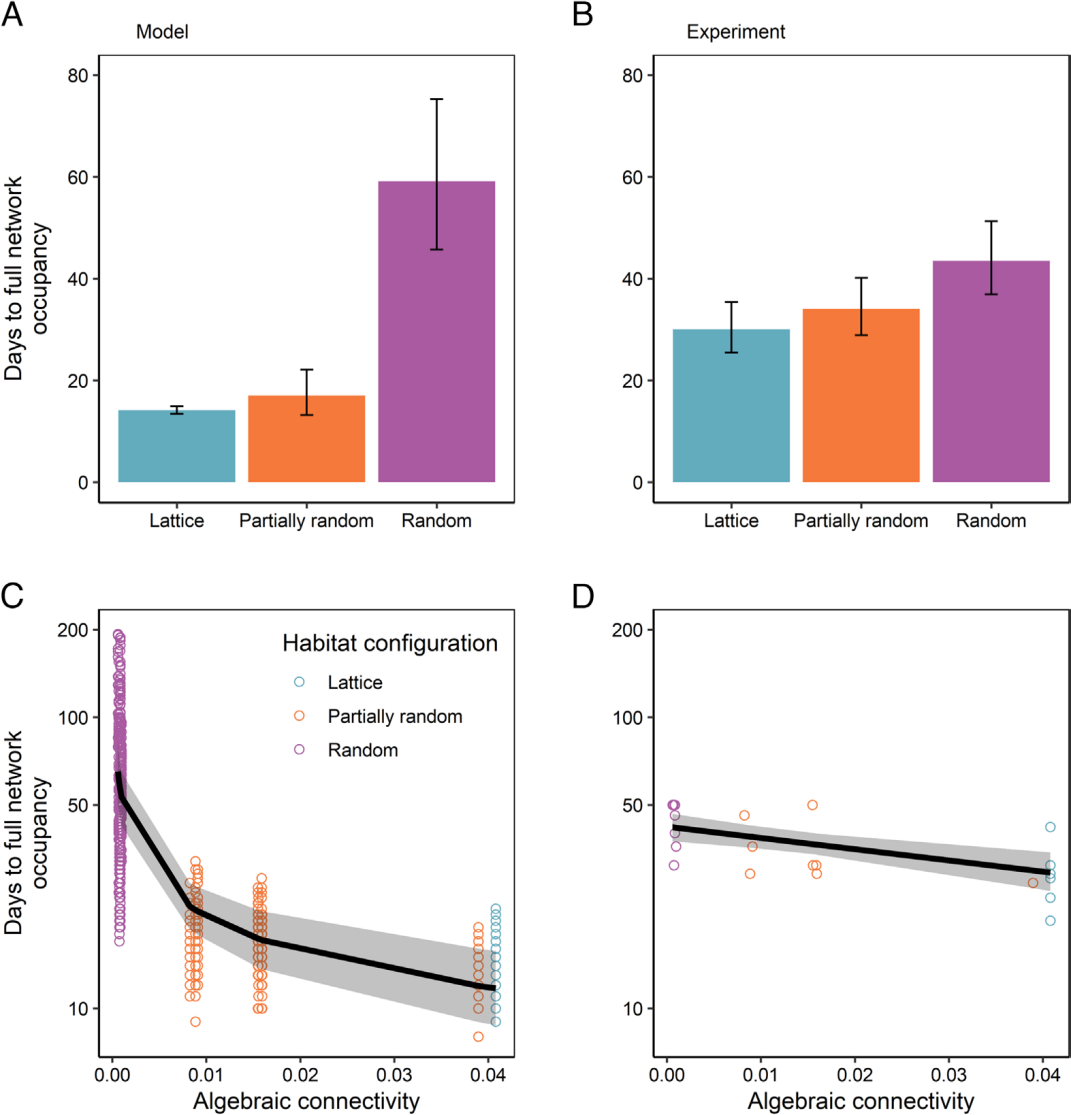
The differences in spread rate among habitat configurations in both the model and the experiment. Time to full network occupancy—number of days until all nodes within a network contained at least one springtail—in each habitat configuration (*A* and *B*) and as a function of the weighted algebraic connectivity of each network (*C* and *D*). Panels on the left (*A* and *C*) show distance-dependent model predictions for *F. candida*, assuming the exponent of the dispersal kernel, *b* = −0.0430; panels on the right (*B* and *D*) show experiment results. Bars and regression lines show predicted values from linear mixed models (*A* and *C*) or linear models (*B* and *D*) with 95% CIs. *Sample sizes:* each model treatment is represented by 50 replicate simulations; each experiment treatment is represented by 8 replicate networks.

Population spread was predicted by habitat network configuration—the configuration of links among habitat nodes—in the model parameterized for the dispersal kernel estimated for *F. candida*. The model predicted that full network occupancy should occur 5.1 and 3.4 times more quickly in lattice and partially rewired networks, respectively, than in random networks (Fig. 2*A*). Time to full network occupancy was predicted to be similar in lattice and partially rewired networks.

The predicted effect of configuration on time to full network occupancy was supported by the results of the experiment: Habitat configuration explained 29% of the variation in time to full network occupancy (ANOVA: F_2,21_ = 5.68, *P* = 0.011; Fig. 2*B*). Spread occurred, on average, 1.4 and 1.3 times faster, in lattice and partially rewired networks, respectively, than in random networks (random vs lattice: t_21_ = 3.22, *P* = 0.011; random vs partially rewired: t_21_ = 2.16, *P* = 0.10). The effect of configuration on colonization rate remained after controlling for differences in link length (*SI Appendix*, Fig. S10). These results, however, are explained by the differences in algebraic connectivity between network configurations.

## Algebraic Connectivity Predicts Time to Full Network Occupancy

Algebraic connectivity was a stronger predictor of spread (time to full network occupancy) than network configuration (lattice, partially random, and random), defined here by the fraction of rewired links in the network (see *SI Appendix*, Tables S2 and S3 for comparison between algebraic connectivity and network diameter). Time to full network occupancy decreased with increasing algebraic connectivity in the model (Fig. 2*C*). The experiment supported this prediction; fully one third (33%) of the variability in time to full network occupancy in the experiment was explained by algebraic connectivity alone (OLS: F_1,22_ = 12.40, *P* = 0.0019; Fig. 2*D*).

Crucially, algebraic connectivity does not increase monotonically with the fraction of rewired links (i.e., randomness; *SI Appendix*, Fig. S11), since due to the geographical embedding of spatial networks, rewiring a link most likely results in changing its length. Algebraic connectivity captures the effect of both changes in link configuration and in link length on spread rate. While the fraction of rewired links does predict days to full occupancy (Fig. 2 *A* and *B*), algebraic connectivity can discriminate between networks with the same fraction of rewired links and is thus a more informative predictor of spread (Fig. 2 *C* and *D*). Therefore, the algebraic connectivity of the dispersal probability network emerges as a metric to measure how easily a species can disperse within a landscape. We now investigate how different dispersal kernels affect the algebraic connectivity of a given configuration, therefore affecting spread rate.

## Spread Rate Depends on Interplay Between Habitat Network Configuration and the Shape of the Dispersal Kernel

**Fig. 3.**
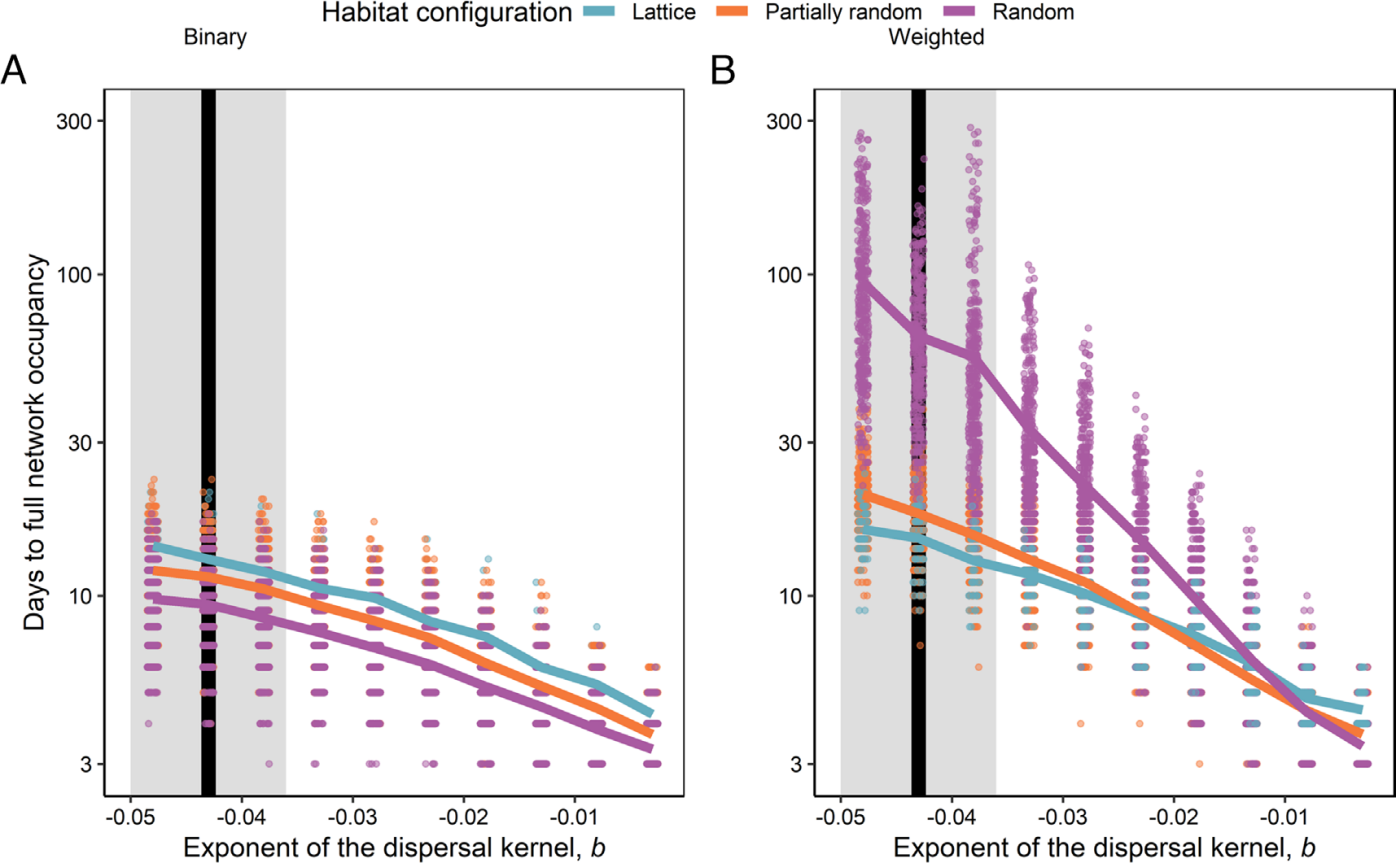
The interaction between habitat configuration and the shape of the dispersal kernel predicts population spread. Model predictions for mean time to full network occupancy as a function of the exponent of the dispersal kernel, *b*, for the model with distance-independent dispersal (*A*) and distance-dependent dispersal (*B*). Each point represents one simulated network (points are jittered to improve visibility). Lines show the bootstrapped mean values for each habitat configuration. The vertical black line and grey shaded area show the estimated value of *b* ± 95% CI for *F. candida*. To make the two panels comparable, the dispersal probability in panel *A* is calculated as the average probability of a given dispersal kernel with exponent *b* (*SI Appendix, Methods*).

When dispersal is independent of distance, that is, when the probability of an individual dispersing between habitat fragments is constant regardless of the distance between those fragments, we see that spread always occurs fastest on random networks and slowest on lattice networks along the full gradient of dispersal probabilities (Fig. 3*A*). Distance independent dispersal is, however, an unrealistic scenario that ignores the fact that movement processes occur in habitats that are geographically embedded.

When dispersal is dependent on distance, according to a dispersal kernel, our model showed that the shape of the dispersal kernel (exponent *b*) interacts with habitat configuration to determine spread rate. The network that promoted the fastest spread changed depending on the value of *b* (Fig. 3*B*). When long-distance dispersal is less likely (*b* is a large negative value), full network occupancy occurred fastest in lattice networks, followed by partially rewired and then fully random networks. In contrast, when long-distance dispersal becomes more likely (when *b* is closer to zero), the ranking between the different network configurations completely reverses. Full network occupancy occurred fastest in fully random networks, followed by partially rewired networks, with lattices being the slowest.

**Fig. 4.**
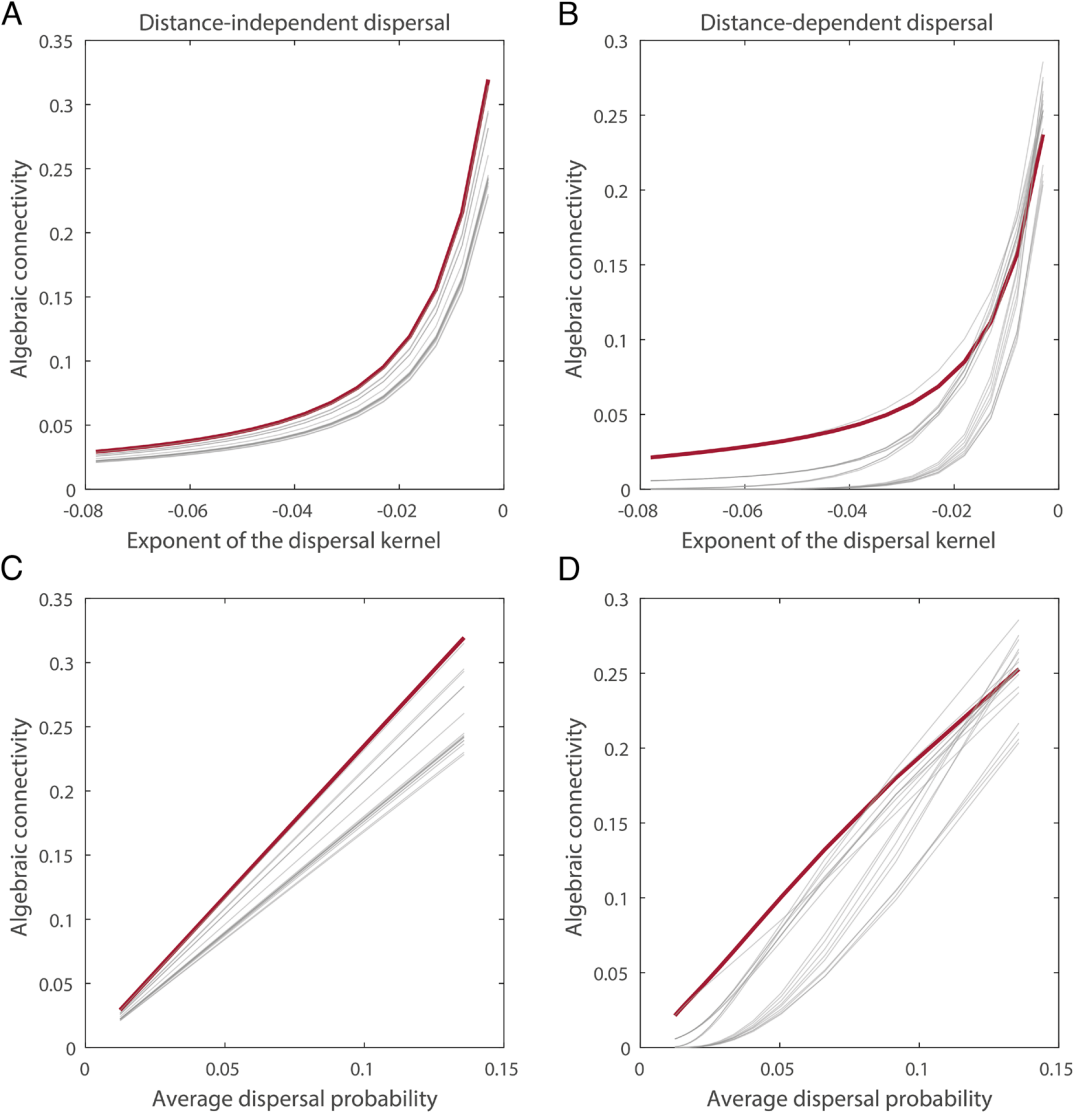
Algebraic connectivity changes as a function of species’ dispersal traits. Each line represents a single network. Algebraic connectivity as a function of the exponent of the dispersal kernel when dispersal is independent of distance (*A*), and when dispersal is dependent on distance – i.e., dispersal probability decays with distance between habitat fragments following the dispersal kernel (*B*). When dispersal probability decays with distance, the rank order of networks depends on the shape of the dispersal kernel. The same result can be observed if algebraic connectivity is plotted against the average dispersal probability (*C* and *D*), as the average dispersal probability is a function of the dispersal kernel (*SI Appendix*, Fig. S7). The networks highlighted in red were chosen to visualize the fact that network ranking stays constant when dispersal is distance-independent, but it changes as a function of the dispersal kernel when dispersal is distance-dependent. This is a general effect that holds true regardless of network size (*SI Appendix*, Fig. S12).

This pattern can again be explained by algebraic connectivity. When we rank networks according to algebraic connectivity, the rank order remains consistent as average dispersal probability increases, as long as dispersal is independent of distance (Fig. 4 *A* and *C*). However, when dispersal is distance-dependent, networks change ranks as average dispersal probability or the shape of the dispersal kernel changes [i.e., a network can flip from facilitating spread to impeding spread, compared to other networks, as the shape of the dispersal kernel changes (Fig. 4 *B* and *D*); this is a general effect that holds true regardless of network size (*SI Appendix*, Fig. S12)]. In other words, the interaction between species’ dispersal traits and habitat configuration can have large effects on population spread, even changing which habitat network configuration is best at promoting spread. The practical consequence of this result is that information about both distance dependence in dispersal (the dispersal kernel) and the configuration of the habitat network is critical for predicting spread rate.

## Discussion

Our results show that connectivity is realized by the dispersal kernel interacting with the habitat network with a particular configuration of nodes and links (47). This means that the habitat network that enables the fastest spread of populations changes depending on the shape of the species’ dispersal kernel. The same networks will have different demographic effects depending on the movement traits of the species considered. This knowledge should guide reserve design and the conservation of multiple species (48).

Algebraic connectivity of the distance-dependent dispersal probability network succinctly encodes the link between the dispersal kernel and the configuration of the habitat and thus is a powerful predictor of spread dynamics. This result corroborates theoretical results from general network theory (39). While previous ecological studies have demonstrated that habitat network configuration influences spread (26, 27, 35), and algebraic connectivity has been theoretically linked to synchronization (49) and coevolutionary dynamics (50), ours provides strong evidence from theory and experiment that algebraic connectivity in weighted networks can predict spatial ecological dynamics. We show that algebraic connectivity can distinguish between networks when the re-wiring fraction of the network is not an extreme value (i.e., not 0 or 1), a condition met by most real-world networks (44). This result suggests that algebraic connectivity is sensitive to small changes in network configuration and is an effective predictor of spread. It is thus a strong candidate metric for predicting ecological dynamics including population spread in fragmented habitats. Tests with data derived from field experiments or movement surveys could validate this prediction in nature.

Species have different perceptions of and responses to the network structure of habitat in the landscape. Consequently, species vary in the impact that habitat configuration has on spread, as their different dispersal kernels are realized on landscapes. We found that when long distance dispersal was rare, spread occurred fastest in lattice networks and slowest in random networks. As the probability of long-distance dispersal increased, the ranked order of configurations flipped such that spread occurred faster in random networks than in lattice and partially rewired networks. This has implications for managing multiple species: a single configuration will not universally facilitate spread equally in all species. This will complicate reserve design and its use, for example, to promote the poleward spread of communities challenged by climate change. Interestingly however, this nonuniversality could be leveraged to preserve native species while discouraging the movement of introduced species (7) as long as their dispersal kernels are different enough.

Our networks contained populations of a single species, and so we have not considered how species interactions may mediate connectivity and spread (51–53). Metacommunity models show that interspecific interactions can strongly impact the speed and success of population spread along environmental gradients (54). Differences in species’ responses to configuration could positively or negatively impact biodiversity, depending on the type of interspecific interaction. Future studies should explore the spread of whole communities including other methods such as algebraic connectivity of multiplex networks (55, 56).

## Conclusion

The major finding of our study is that dispersal behavior, captured by the dispersal kernel, and habitat network configuration jointly predict the rate of spread of populations occupying networks of fragmented habitat; the network that best facilitates spread cannot be determined by solely knowing either the dispersal ability or the habitat configuration. We found a strong relationship between population spread and algebraic connectivity, which captures essential information about how the dispersal kernel (species-specific dispersal distances) is realized on habitat networks. We recommend that future studies of population processes on spatial habitat networks either control for distance or use connectivity metrics, like algebraic connectivity, incorporating information about the dispersal kernel. Our findings may be used to improve conservation outcomes, especially when modifying habitat connectivity is part of an integrated strategy to protect species in fragmented landscapes.

## Materials and Methods

### Network Design.

Each network was comprised of 10 nodes and 20 links. Within a network, the 10 nodes were fixed in a ring with 15 mm between neighboring nodes (node edge–node edge). In lattice networks, each node was connected to its two nearest neighboring nodes via 15-mm links, and to its two second-nearest neighboring nodes via 90-mm links (Fig. 1). For partially rewired and random networks, we started with hypothetical lattice networks. Each link then had a probability of being “rewired” to a randomly selected node. Rewiring probability was 20% for partially rewired networks and 100% for random networks. Since node placement was fixed, rewiring had the potential to change the length of the link required to span the distance between nodes. Link length ranged from 15 mm to 190 mm in both partially rewired and random networks, with mean ± SD link length 65 ± 3 mm and 120 ± 8 for partially rewired and random networks, respectively. We created eight versions of partially rewired networks and eight versions of random networks (variation in topology is impossible for lattice configuration), for a total of 17 unique network topologies. Complete information detailing each unique network topology is provided in the shared data.

### Theory.

To explore the interplay between dispersal and habitat network configuration, we used a stochastic individual-based model based on the life history of the model organism *Folsomia candida*. Life history parameter estimates were taken from (ref. (42). The model was run on the 17 spatial networks described above, with 50 replicate simulations on each network. At the beginning of the simulation, the network was empty but at the first time step, 200 individuals were randomly assigned among the four size classes and added to the source patch *S* (Fig. 1*C*). For each subsequent time step, we first calculated the probability that individuals reproduce, then disperse, and then die. Each time step was considered to have a length of 1 d. The model duration was 200 d.

We created two versions of the model: a distance-dependent and a distance-independent version. In the distance-dependent version, dispersal probability was a function of the distance between two nodes. Based on empirical information (*SI Appendix*), we assumed that the dispersal kernel followed a negative exponential function. The dispersal probability of an individual between two patches *j* and *k*, was calculated as follows:

[1] $$D_{j,k}=a\;\cdot \;exp\;(b\;\cdot \;W(j,k)),$$

where $W\left(j,,,k\right)$ is the distance between those two nodes, a is the probability of moving from one node to another when the distance between two nodes is zero, and *b* is the exponent that controls the shape of the dispersal kernel (how fast the dispersal probability decays with distance, Fig. 1*B*). In the distance-independent version—to draw fair comparisons between the outputs of the distance-dependent dispersal and distance-independent dispersal models for a given value of *b*—the dispersal probability between any two adjacent nodes in the network is equal to the average dispersal probability of the kernel with that specific exponent (*SI Appendix*, Fig. S7). To obtain this average value, the kernel function is evaluated between 15 and 190 mm (minimum and maximum distance in our experimental setup).

In both versions of the model, an individual in patch *j* moves to a neighboring patch *k* if a random number drawn from a uniform pseudorandom distribution is equal or smaller than $D_{j,k}$ . To give all neighboring patches an equal chance to receive individuals, we randomized the order in which we iterated through the neighboring patches. Only individuals older than 7 d dispersed; we assumed that juveniles did not disperse, and that the dispersal probability does not depend on the age of the individual once it is no longer a juvenile. We also assumed that dispersal operates on a faster time scale than demography, so when an individual moved, it remained in the same age class. Full details on model methods can be found in *SI Appendix*.

### Experiment.

To test the predictions of the theoretical model, we created physical habitat networks for *F. candida* to inhabit that matched the 17 networks described above and used in the theoretical model. We created all 17 unique network topologies and replicated the lattice configuration eight times. All habitat configurations (lattice, partially rewired, random) were therefore represented by eight physical replicate networks in the experiment, for a total of 24 networks. Habitable nodes in these networks were cylindrical vials with a base of an artificial soil substrate. Nodes were linked using tubes but these were not habitable and acted only as movement corridors. To begin the experiment, we placed 10 randomly selected collembola into node *S* of each network (Fig. 1*C*). To estimate population sizes, we photographed each node typically every 1 to 2 (maximum 5) d, and then used automated image-processing analysis to count the number of individuals in each photograph. The experiment lasted a total of 182 d. For a full description of the experiment methods see *SI Appendix*.

## Supplementary Material

Appendix 01 (PDF)

Dataset S01 (XLSX)

## Data Availability

All code and data can be downloaded from the permanent institutional repository Eawag Research Data Institutional Collection (https://doi.org/10.25678/0005V2), and from GitHub (https://github.com/GonzalezBiodiversityLab/Springtail-networks).
