# Supplementary material for: Spread of networked populations is determined by the interplay between dispersal behavior and habitat configuration
Source: Proc Natl Acad Sci U S A. 2023 Mar 9;120(11):e2201553120. doi: 10.1073/pnas.2201553120 (PMC10089154; doi:10.1073/pnas.2201553120)
Supplement: Supplementary file 1 — Appendix 01 (PDF) [file pnas.2201553120.sapp.pdf]

Supplementary Information for

SPREAD OF NETWORKED POPULATIONS IS DETERMINED BY THE INTERPLAY BETWEEN DISPERSAL BEHAVIOR AND HABITAT CONFIGURATION

Bronwyn Rayfield[1†‡], Celina B. Baines[1†], Luis J. Gilarranz[2†], and Andrew Gonzalez[1*]
† Equal co-authors

1. Department of Biology, McGill University, 1205 Dr. Penfield Ave., Montreal, QC, H3A 1B1, Canada
2. EAWAG: Swiss Federal Institute of Aquatic Science and Technology, Überlandstrasse 133, 8600 Dübendorf, Switzerland

‡ Present address: Apex Resource Management Solutions Ltd., 937 Kingsmere Ave., Ottawa, ON, K2A 3K2, Canada
*Author for correspondence: Andrew Gonzalez, **Email:** andrew.gonzalez@mcgill.ca

This PDF file includes:

Supplementary methods
Supplementary results
Table S1-S3
Figures S1-S12
SI References

Supplementary Methods

S1. Characterization of spatial networks as a function of rewiring

The networks used in this study represent habitat fragments linked by dispersal corridors. By studying fragmented habitats as networks, we can characterize and analyze their structure and relate that analysis to the observed node and network population dynamics.

All networks were comprised of 10 nodes and 20 links. Within a network, the 10 nodes were fixed in a ring with 80 mm (node centroid – node centroid) between neighboring nodes (Fig. S1). We used three types of networks of varying degrees of link heterogeneity. In 'lattice' networks, each node was connected to its two nearest neighboring nodes and to its two second-nearest neighboring nodes. For the second type of network, we randomly rewired 20% of the links, in the same way Watts and Strogatz (1) created their 'small world' networks. We call these partially rewired networks since these networks are of small size (small number of nodes and links) and so did not uniformly have a small world structure. Finally, we created a third group of networks we call 'random' by randomly rewiring all of the links.

All of the lattice networks necessarily have the same topology (i.e., same links between nodes), while we used eight different partially rewired network topologies, and eight different random network topologies, for a total of 17 unique network structures. Since node placement was fixed, rewiring had the potential to change the length of the link required to span the distance between nodes (Fig. S1). In lattice networks, each node was connected to its two nearest neighbors via 15 mm links and to its two second-nearest neighbors via 90 mm links. Link length ranged from 15 mm to 190 mm in partially rewired and random networks. Mean ± s.d. link length was 65 ± 3 mm and 120 ± 8 for partially rewired and random networks, respectively. Information detailing the structure of each network is provided in the data repository: https://doi.org/10.25678/0005V2.

S2. Study organism

This study was conducted with the microarthropod *Folsomia candida* Willem (Collembola: Isotomidae). *Folsomia candida* dwells in soils worldwide in a variety of habitats including forests, stream banks, agricultural systems, and moss (2). They feed on fungi, including plant root mycorrhizae (3). Our population reproduced by parthenogenesis and therefore contained only females. Individuals start to oviposit three weeks after hatching and the eggs hatch after one week. The lifespan of an individual is approximately 111 days at 25 ºC. Adult *F. candida* are approximately 1.5 – 3.0 mm in length (2).

S3. Theoretical model

We created a stochastic agent-based version of the Markov-chain model introduced in Gilarranz et al. (4), and parameterized following Beddington (5). This stochastic version of the model allows us to better explore the role of network and node topological characteristics in the spread of populations. It is particularly useful when the number of individuals is small and the local populations are far from their carrying capacity.

The model was run on the spatial networks described above (*S1. Characterization of spatial networks as a function of rewiring*). The model was run 50 times in each of the 17 different network structures. In each of those 50 model replicates, we use different initial conditions. Those initial conditions are different across replicates but the same across network topologies. At the beginning of the simulation, the network is empty. In the first time-step, a population of 200 individuals randomly assigned between the four size classes is inoculated into node 5. For each subsequent time step, we first calculate the probability that each individual reproduces, then disperses, and then dies. Each time step is considered to have a length of one day.

Survival

Density-dependent survival, $S(N)$, is calculated as follows:

$$S(N) = (d - e\ln(N))/7 \qquad \text{(Eq. S1)}$$

where d = 1.35, and e = 0.14. The upper-limit of survival was set to one. Both reproduction and survival probabilities were parameterized according to Beddington (5). Individuals die if they reach their maximum life span of 111 days estimated empirically at 25 ºC (2).

Reproduction

Reproduction probability of an individual i is a function of the total number of individuals in the node where that individual is at that time step (Eq. S2). Only individuals of the two largest classes – individuals which are at least 29 days old – reproduce. Density-dependent reproduction, $F(N)$, is calculated as following:

$$F(N) = (p\ln(N) + q\ \ln(N)^2 + c)/7, \qquad \text{(Eq. S2)}$$

where N is the population size of the node, p = 18.53, q = -1.74, and c = -44.04. We divide by 7 to obtain a daily rate. The lower-limit of the probability was set to zero. For each individual at least 29 days old, we draw a random number obtained from a uniform pseudorandom distribution. If the random number is smaller than the reproduction probability, then we add a new juvenile to that node.

Dispersal

Dispersal probability is a function of the distance between two nodes. We assume that the dispersal kernel follows a negative exponential function. The dispersal probability of an individual between two nodes j and k, is calculated as follows:

$$D_{j,k} = a \cdot \exp(b \cdot W(j,k)), \qquad \text{(Eq. S3)}$$

where $W(j,k)$ is the distance between those two nodes, a is the probability of moving from one node to another when the distance between two nodes is zero, and b is the exponent that controls the shape of the dispersal kernel (how fast the dispersal probability decays with distance, Fig. 1*B* in the main text). In the distance-dependent dispersal model, an individual in node j moves to a neighboring node k if a random number drawn from a uniform pseudorandom distribution is equal or smaller than $D_{j,k}$. In order to give all connecting nodes an equal chance of receiving individuals, we randomize the order in which we iterate through the connected nodes. Only individuals older than 7 days disperse, meaning that we assume that juveniles do not disperse, and that the dispersal probability does not depend on the age of the individual once it is no longer a juvenile. We also assume that dispersal operates on a faster time scale than demography, so when an individual moves, it remains in the same age class. When dispersal is considered to be distance-independent,

in order to compare distance-dependent dispersal and distance-independent dispersal for the same value of the exponent b, the values $w_{i,j}$ are all equal to the average dispersal probability of the kernel with that specific exponent (Fig. S7). To obtain this average value, the kernel function is evaluated between 15 and 190 mm (minimum and maximum distance in our experimental setup).

S4. Experiment Methods

S4.1. Network construction

We created habitat networks each comprised of 10 habitat patches (nodes) connected via corridors (links) that allowed individuals to move between habitat patches (Fig. S1). Habitat patches were polypropylene vials (30 mm diameter, 85 mm height) 2/3 full of an artificial soil substrate (a mixture of de-ionized water and activated charcoal:plaster of Paris in a 1:8 ratio by weight). Holes were pierced in the lid of each vial to enable air exchange; the lids were always closed except when vials were being photographed. We burned holes into the sides of each vial at the level of the surface of the artificial soil substrate, and then inserted Tygon tubing (6 mm outer diameter, 4.4 mm inner diameter) to act as links. These links were not habitable but acted only as movement corridors. The tubing was attached to the vials using connectors (wide ends of pipette tips – Eppendorf epT.I.P.S. – cut to 15mm in length) glued into the holes in the sides of the vials.

The links were attached to the nodes to create the 17 network structures described above (*S1. Characterization of spatial networks as a function of rewiring*). Partially rewired and random configurations were each represented by eight physical networks (each with different topologies but the same rewiring probability). We also created eight replicate lattice networks. We therefore had a total of 24 physical networks.

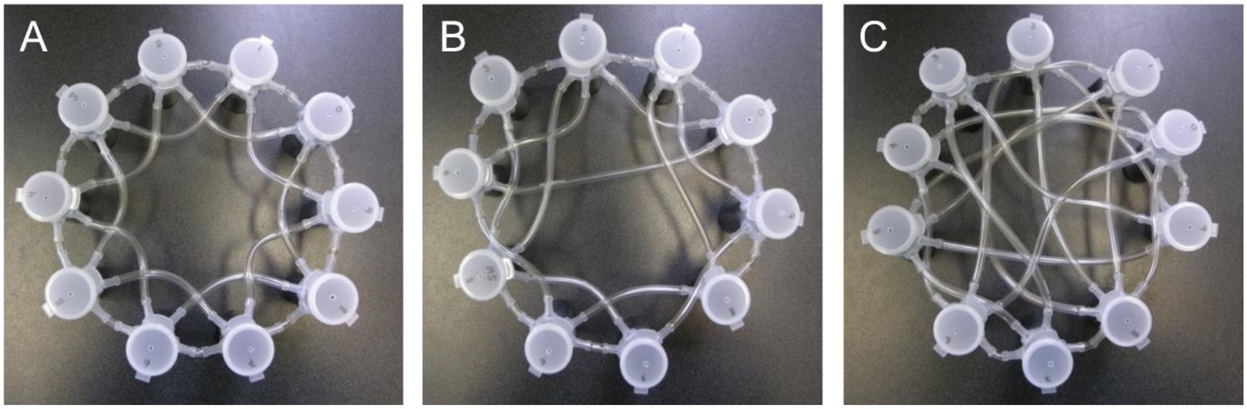

Figure S1. Photographs from the experiment of representative lattice (A), partially rewired (B), and random (C) networks.

S4.2. Experiment maintenance

The experimental animals used in this study were obtained from a laboratory culture from Environment Canada, which were taxonomically confirmed as *F. candida*. The culture was acclimated to a temperature of 20 ± 2°C; the lab was maintained at this temperature for the duration of the experiment. De-ionized water was sprayed onto the artificial soil substrate once per week during the experiment, to maintain moist but not waterlogged conditions. Food (granulated dry baker's yeast) was added to each node following a unique, autocorrelated feeding regime *sensu* Gonzalez and Holt (6). This created spatiotemporally variable resource heterogeneity to encourage movement among the nodes. Every other day of the experiment, each node was defined as being in one of two states: 'food added' or 'no food'. We started by randomly assigning 30% of the nodes to the 'food added' state on day 0; the rest of the nodes were assigned the 'no food' state. Every two days, each node was given a probability of transitioning between states, governed by the transition matrix, *T*:

$$T = \begin{pmatrix} 1-\lambda & \lambda \\ \lambda & 1-\lambda \end{pmatrix} \quad \text{(Eq. S4)}$$

where $0 < \lambda < 1$ and λ is the probability of a change in state between a day and the day two days preceding it. We set $\lambda = 0.2$, meaning that food availability exhibited positive temporal autocorrelation. If a node was in a 'food added' state, we added 1 granule of baker's yeast; if a node was in a 'no food' state, no yeast was added. Old, uneaten food was removed prior to adding new food. Following this feeding regime, food was added to each node an average of 45 ± 0.74 days over the course of the 182-day experiment.

S4.3. Data collection

To begin the experiment, we placed 10 randomly selected collembola into node 5 of each network. To estimate population sizes throughout the experiment, we photographed each node typically every 1-2 (maximum 5) days (Fig. S2*A*). The duration of the experiment was 182 days; photographs were taken on 97 days of the experiment. Photographs were in jpeg format, captured in RGB, and were 3264 × 2448 pixels and 30 MB in size. This generated ~45000 photographs. Since it was not feasible to process (count all the collembola in) all of these photographs manually, we developed an algorithm that automatically estimated the number of individuals in each image (see section *S5. Image recognition analysis*).

S5. Image recognition analysis

We developed an algorithm in MATLAB to estimate the number of collembola in each node. First, each jpeg (Fig. S2*A*) was converted to grayscale, scaled between 0 and 255. The surface of the node was then isolated through a routine that converged on the center of the dark node surface and identified the radius of the node through iteration. The image was then cropped to the boundaries of the node surface (Fig. S2*B*). Visual noise in the image caused by variation in the color of the artificial soil surface was removed by converting the image to a binary (black and white) image (Fig. S2*C*). The algorithm then identified and outlined discrete white objects on the black background, using functions from the Image Processing Toolbox in MATLAB.

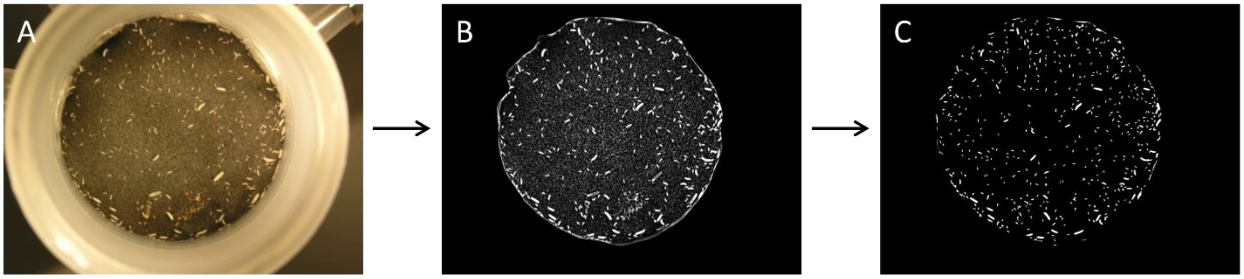

Figure S2. Illustration of the steps taken in the image recognition analysis. A) An example of an original image. Collembola of various sizes as well as pieces of yeast food are visible. B) The image is cropped to the boundaries of the node. C) Visual noise is removed, making the collembola more visible.

In order to distinguish collembola from other white objects in the node (e.g., food, eggs, debris), we compared the size and shape of objects identified by the algorithm to a reference distribution of real collembola. The reference distribution was built using 500 manually identified collembola from 16 photos of individual nodes. We used functions from the Image Processing Toolbox in MATLAB to measure the aspect ratio (body length / body width), area, and perimeter to area ratio (measured in pixels, see Table S1 for formulae) of each of the white objects. Area was adjusted to account for different zoom levels/camera heights by estimating it relative to the radius of the node (Table S1). We then set lower and upper cutoff values for each of these parameters as the 2.5th and 97.5th quantiles of their distributions, with the exception that the lower cutoff for area was set to 100 based on manual measurements of original photos (Table S1).

Table S1. Formulae and cutoff values for each of the size and shape parameters used to distinguish real collembola from other objects in the nodes.

Parameter	Formula*	Lower cutoff value	Upper cutoff value
Aspect ratio	L/w	2.7	5.0
Area (adjusted)	$\left(1000/10^6 \cdot r\right)^{2A}$	100	2000
Perimeter to area ratio	$P/2 \cdot \sqrt{\pi A'}$	1.2	1.9

* L : body length, w : body width, r : node radius, A, A' : area of body (unadjusted, adjusted), P : perimeter of body

We excluded any object identified in the experimental images that did not fall within the cutoff values for all parameters. Population size was then estimated as the number of collembola-shaped objects in the node. We validated the image recognition analysis by manually counting individuals in 30 images and comparing these "true" counts to the estimates generated by the algorithm. These values were highly correlated ($r = 0.93$, $R^2 = 0.86$; Fig. S3). We see that estimated counts tend to underestimate the true counts, particularly at high abundances (range 150-250 individuals). However, the high correlation validates the use of our automated image processing procedure. Note that, although effective, the methods described above have now been updated by the methods described by Gilarranz et al. (4).

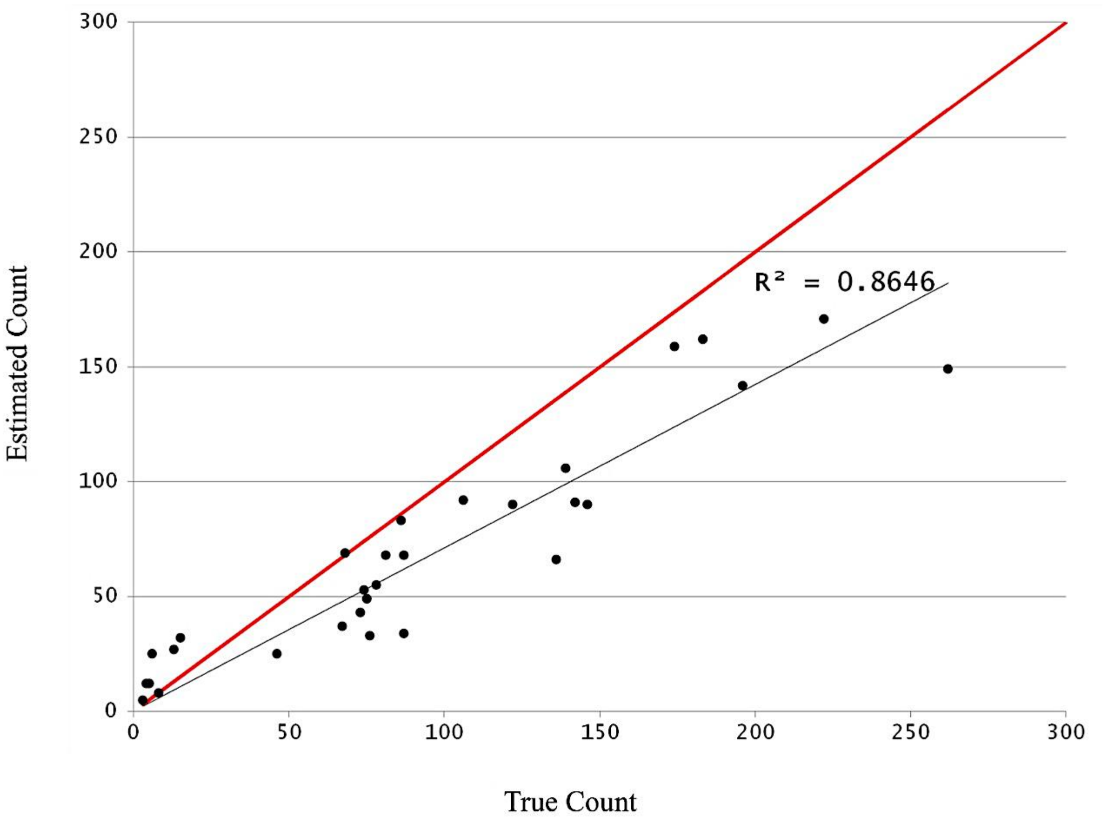

Figure S3. Results of the image recognition analysis validation. Estimated counts of collembola generated by the algorithm are regressed on "true" counts of collembola taken manually. The estimated and true counts are highly correlated. The 1:1 line is shown in red.

S6. Estimation of the *Folsomia candida* dispersal kernel

The dispersal probability as a function of distance (Fig. 1*B* in the main text) was calculated in a separate pilot experiment (Fig. S4). For this experiment we had two-node networks linked by tubes of varying lengths: 15, 90, 130, 170, and 190 mm, reproducing all the lengths that we find in the networks used for the main experiment (nodes and links are described in section *S4.1. Network construction*). We used five replicates of each distance. One of the nodes (source) in each pair was inoculated with exactly 84 adult individuals, and the number of individuals was tracked daily in both source and destination nodes for 20 days, while keeping them fed with a pattern resembling that of the main experiment.

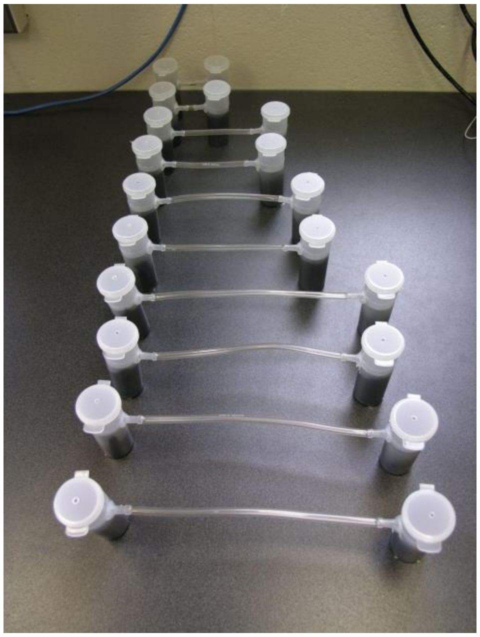

Figure S4. Experimental setup to obtain the dispersal probability as a function of distance. Two-nodes networks linked by tubes of varying lengths (15, 90, 130, 170 and 190 mm). Only one of the two nodes was inoculated. Population sizes were tracked in both nodes daily for a 20-day period.

In Fig. S5 we show the number of individuals that disperse between the two nodes each day, obtained by calculating changes in population sizes in the number of adults in either node (source or destination).

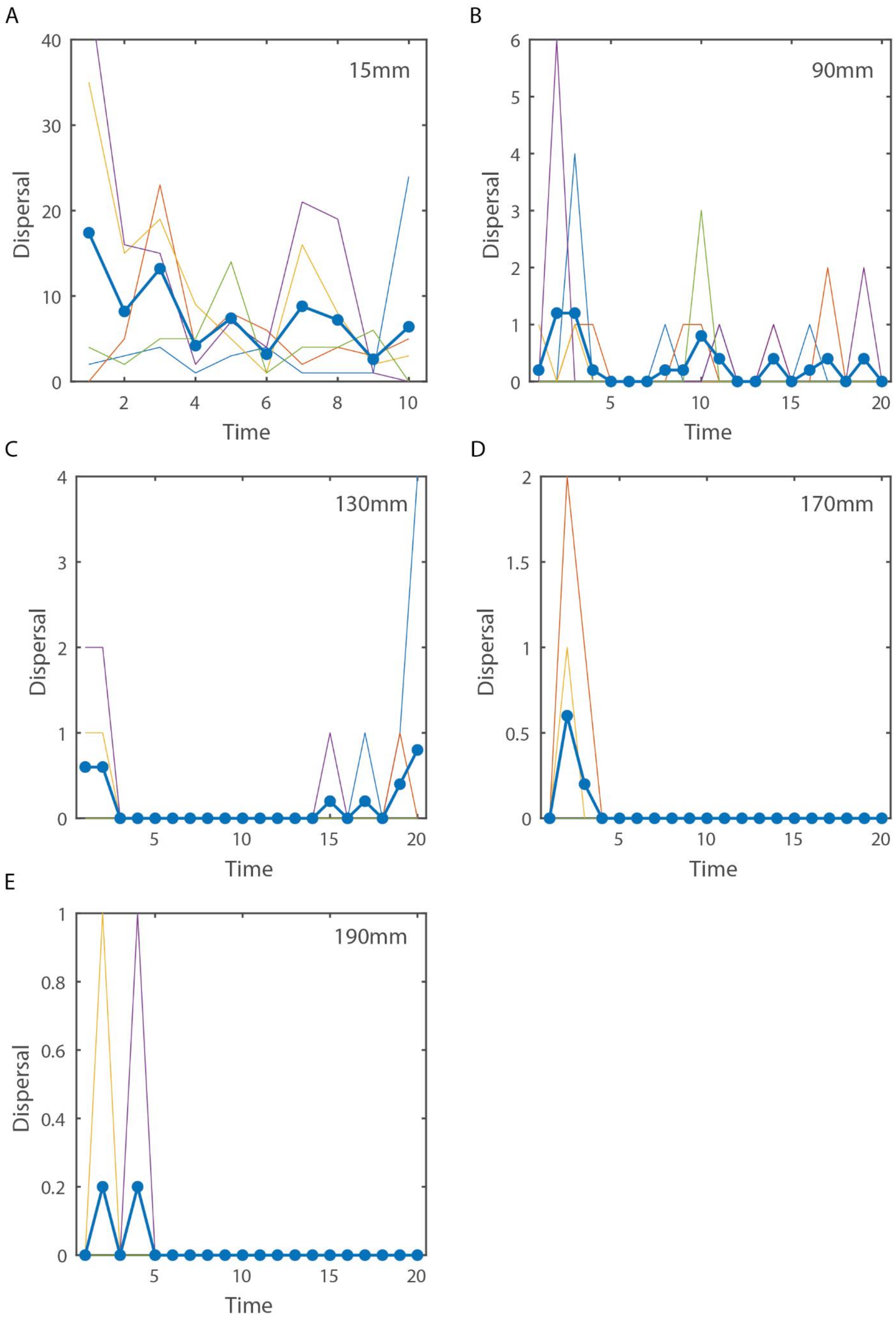

Figure S5. Time series of the number of individuals that disperse between two nodes. Thin coloured lines represent each individual replicate. Thick blue lines represent the average across all five replicates. Each panel shows one of the target distances–shown in the panel label at the top-right corner.

Since the population of adult individuals remains constant for the length of this experiment, it is straightforward to transform the number of individuals that disperse between two habitat

fragments, into dispersal probability. The probability of a single individual dispersing from one patch to an adjacent patch linked by a given distance is the average number of individuals that dispersed at that distance on a day for all the replicates, divided by the size of the population in the two-patch network (84 individuals). In Fig. S6 we show the individual dispersal probability as a function of the distance between two habitat fragments (Fig. S6).

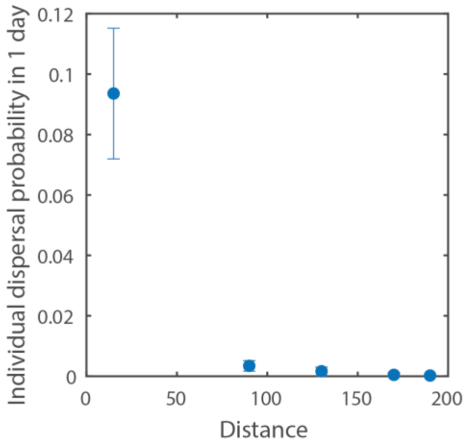

Figure S6. Probability of a single individual dispersing in one day between two habitat fragments as a function of the distance between said fragments. The error bar shows the standard error between replicates. Distance is given in mm.

The individual dispersal probability in 24h ($D_{i,j}$) as a function of distance between habitat fragments ($w_{i,j}$) in this pilot experiment can be modelled as a negative exponential, following the form:

$$D_{i,j} = a \cdot exp(b \cdot w_{i,j}), \quad \text{(Eq. S5)}$$

where a = 0.178 $\pm$ 0.019, and the exponent b = -0.043 $\pm$ 0.007. The R^2 between the empirical dispersal data and this negative exponential model was 0.9998.

Finally, we explored whether there were any signs of density-dependent dispersal by searching for evidence of a non-linear relationship between the change in the number of individuals in one node as a function of the number of individuals in the other node. We found no evidence to support density-dependent dispersal.

S7. Spatial properties of nodes and networks

S7.1. Minimum distance from source

For each node, the minimum distance from the source is the length, in millimeters, of the shortest path between that node and node 5 or "source node", the node in which we placed collembola at the beginning of the experiment/model simulation.

S7.2. Algebraic connectivity

Algebraic connectivity is defined as the second-smallest eigenvalue of the normalized Laplacian matrix (7). The elements $l_{i,j}$ of the normalized Laplacian matrix L of a weighted graph W are defined as:

$$l_{i,j} = \begin{cases} 1, & \text{if } i = j, \text{and } w_{i,j} \neq 0 \\ -1/\sqrt{s_i \cdot s_j}\,, & \text{if } i \neq j, \text{and } i \text{ is adjacent to } j, \\ 0, & \text{if } i \neq j, \text{and } i \text{ is not adjacent to } j \end{cases} \qquad \text{(Eq. S6)}$$

where s_i and s_j are the strengths of nodes i and j respectively.

The elements $w_{i,j}$ of the graph W represent the probability of an individual to disperse between nodes i and j in 24 hours. When dispersal is considered to be distance-dependent, that probability is given by the dispersal kernel (Fig 1*B* in the main text). The shape of the kernel, and therefore the values of $w_{i,j}$ are controlled by the exponent b (Eq. S5). When dispersal is considered to be distance-independent, the values of $w_{i,j}$ still depend on the exponent b, so that the outputs of the distance-dependent dispersal and the distance-independent dispersal models are comparable. However, in the distance-independent version, all values of $w_{i,j}$ are the same for a given value of b. Fig. S7 shows the relationship between the exponent of the dispersal kernel and the $w_{i,j}$ values in the distance-independent model.

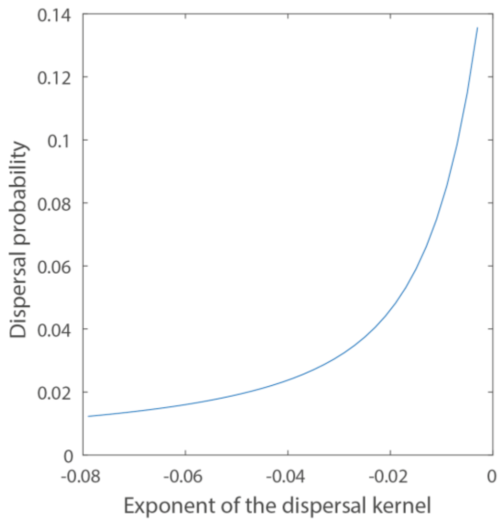

Figure S7. Relationship between the exponent of the dispersal kernel and the average dispersal probability of the kernel when dispersal is distance-independent.

S8. Statistical analysis

S8.1. Random effects in analysis of spread

We had to account for several random effects in our analysis of the effects of habitat network configuration and spatial properties on spread. In the model, topology was used as a random effect when considering partially rewired and random networks, because we expect some variance in time to colonization and time to full network occupancy to be explained by the exact topology of the network, and each network topology was replicated 50 times. All lattice networks had the same topology, and so this random effect was not included in the analysis of lattice networks. Model replicate (repID) was also included in the analysis of time to node colonization, to account for the non-independence of nodes within the same simulated network. When analyzing time to node colonization in the experiment, we also accounted for the non-independence of nodes within the same physical network; this variable is named landscapeID.

S8.2. Time to node colonization and full network occupancy

Estimating time to node colonization and full network occupancy

We investigated spread at the node and network levels. Spread at the node level was measured as the time to colonization, estimated as the first day on which at least one individual was observed in that node. We excluded node 5 of each network from all analyses of colonization because we placed collembola in this node at the beginning of the experiment and so it was not naturally colonized. Spread at the network level was measured as the time to full occupancy, estimated as the day on which every node in the network had been colonized. All analyses were conducted in R v4.0.2. (8).

Effects of habitat network configuration on spread

We explored the effects of habitat network configuration on spread in the model parameterized with the dispersal kernel for *F. candida* and in the experiment with *F. candida.* To analyze this using model data, we built random effects models for partially rewired and random network configurations in the R package 'lme4' (9). We used log10-transformed time to full network occupancy as the response variable and included network topology as a random effect. For lattice networks, we build an intercept model with log10-transformed time to full network occupancy as the response. Mean time to full occupancy for each configuration was estimated using a bootstrapping procedure with 1000 bootstrap replicates, using the ‘boot’ function in the R package ‘boot’ (10, 11). 95% confidence intervals were estimated as the range from the 2.5^{th} and 97.5^{th} percentiles of the bootstrapped sample values.

We tested the effect of habitat network configuration on time to full network occupancy in the experiment using a linear fixed-effects model with log10-transformed time to full occupancy as the response and habitat network configuration included as a fixed effect. We used this model to estimate the mean time to full network occupancy ± 95% confidence intervals for each network configuration. We used the ‘anova’ function in R to conduct an overall significance test. Habitat network configuration had a significant effect on time to full network occupancy in the experiment, so we used Tukey post-hoc tests to contrast each pair of network configurations.

Effects of spatial metrics on spread

We investigated whether spread could be predicted by metrics describing the spatial properties of nodes and networks in the model parameterized for *F. candida* and in the experiment. At the node level, we investigated minimum distance from source. To analyze model data, we built linear mixed models with log10-transformed time to node colonization as the response variable. We included habitat network configuration, the second-degree polynomial of distance from source, and their interaction as fixed effects. Network topology and model replicate (repID) were included as random effects. We conducted a bootstrapping procedure of model estimates (1000 replicates) to estimate the mean time to node colonization ± 95% confidence intervals (2.5^{th} and 97.5^{th} percentiles of bootstrapped sample values) for each network configuration and as a function of minimum distance from source.

To analyze the empirical data, we built a linear mixed model with time to node colonization as the response. Fixed effects were minimum distance from source, habitat network configuration, and their two-way interaction. The identity of the physical network (landscapeID) was included as a random effect. We conducted a bootstrapping procedure of model estimates (1000 replicates) to estimate the mean time to node colonization ± 95% confidence intervals (2.5^{th} and 97.5^{th} percentiles of bootstrapped sample values) for each network configuration and as a function of minimum distance from source. We used the 'drop1' function in R to conduct a significance test.

At the network level, we investigated the effect of weighted algebraic connectivity on time to full network occupancy. To analyze model data, we built a linear mixed model with log10-transformed time to full occupancy as the response, and log10-transformed weighted algebraic connectivity as a fixed predictor. Topology was included as a random effect. We used a bootstrapping procedure with 1000 replicates to estimate the mean ± 95% confidence intervals (2.5^{th} and 97.5^{th} percentiles of bootstrapped sample values) of the time to full network occupancy as a function of weighted algebraic connectivity.

To analyze experiment data, we used a linear fixed-effects model with log10-transformed time to full occupancy as the response, and weighted algebraic connectivity included as a fixed effect. We used a bootstrapping procedure with 1000 replicates to estimate the mean ± 95% confidence intervals (2.5^{th} and 97.5^{th} percentiles of bootstrapped sample values) of the time to full network occupancy as a function of weighted algebraic connectivity. We used the 'anova' function in R to conduct a significance test.

Effect of the shape of the dispersal kernel on spread

We tested for the effect of the exponent of the dispersal kernel, *b*, on spread in the model. We obtained bootstrapped estimates (1000 replicates) of time to full network occupancy for each configuration and as a function of *b*.

Supplementary Results

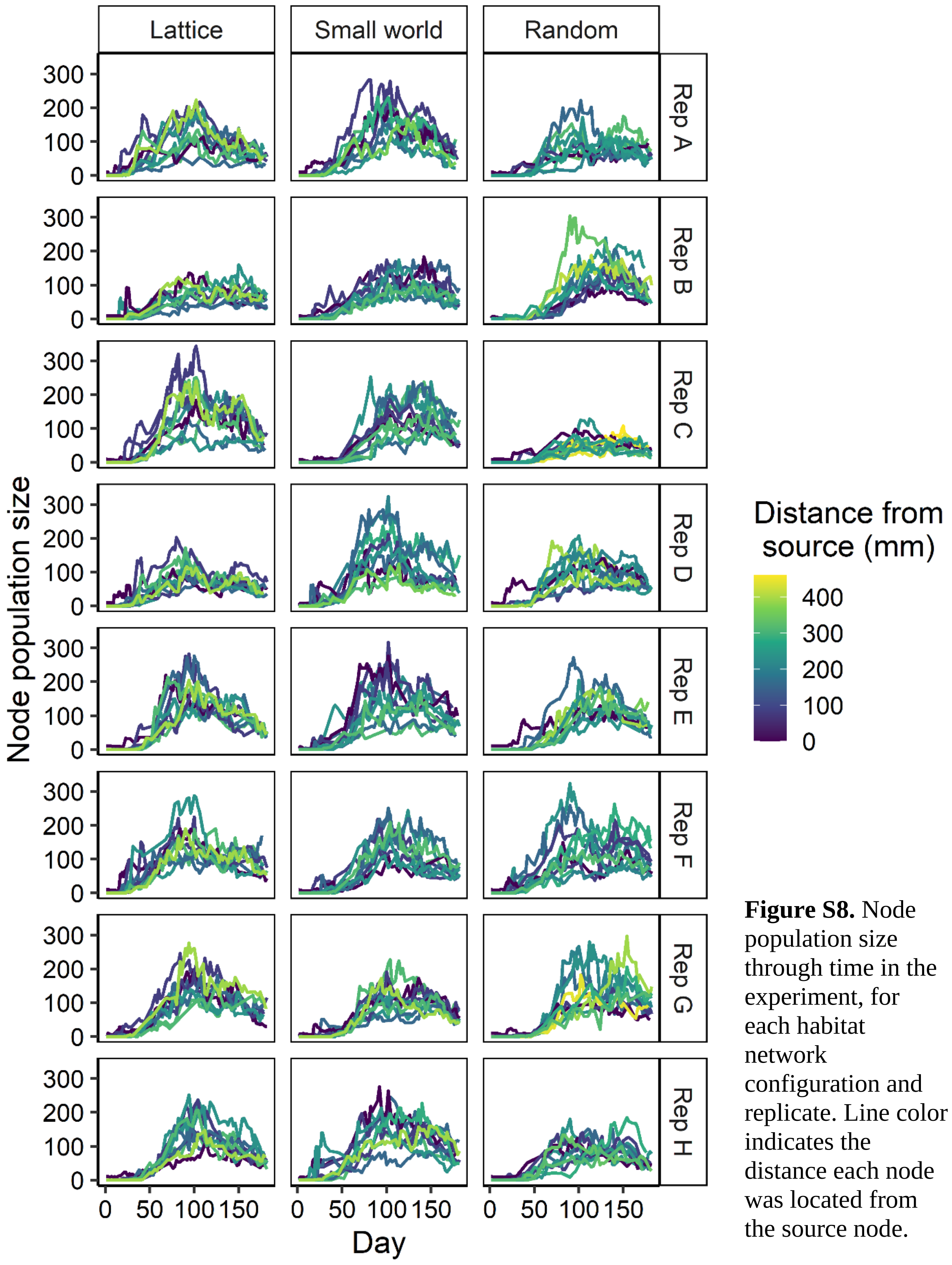

Figure S8. Node population size through time in the experiment, for each habitat network configuration and replicate. Line color indicates the distance each node was located from the source node.

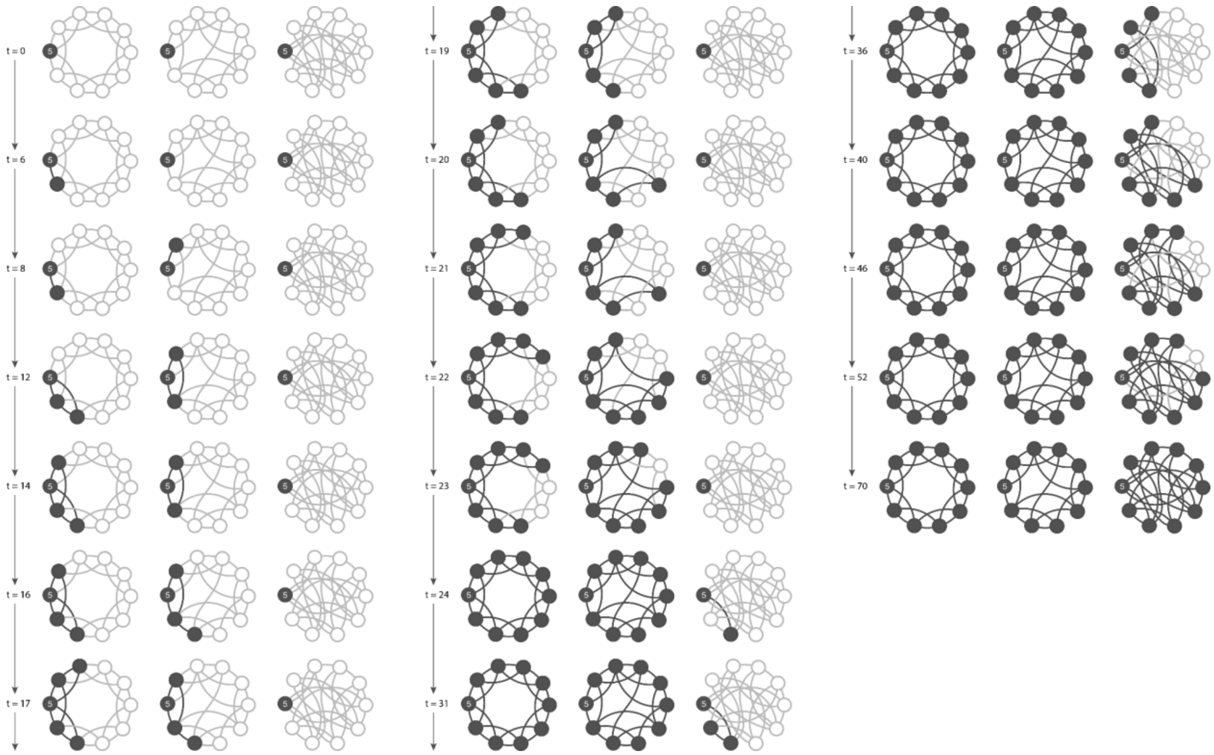

Figure S9. From inoculation to full network occupancy. For the same empirical networks shown in Fig. 1 in the main text (from left to right of each column: lattice, partially rewired, and random), here we show the colonization process observed in the experiment for these particular replicates. Black nodes are occupied and white nodes are empty.

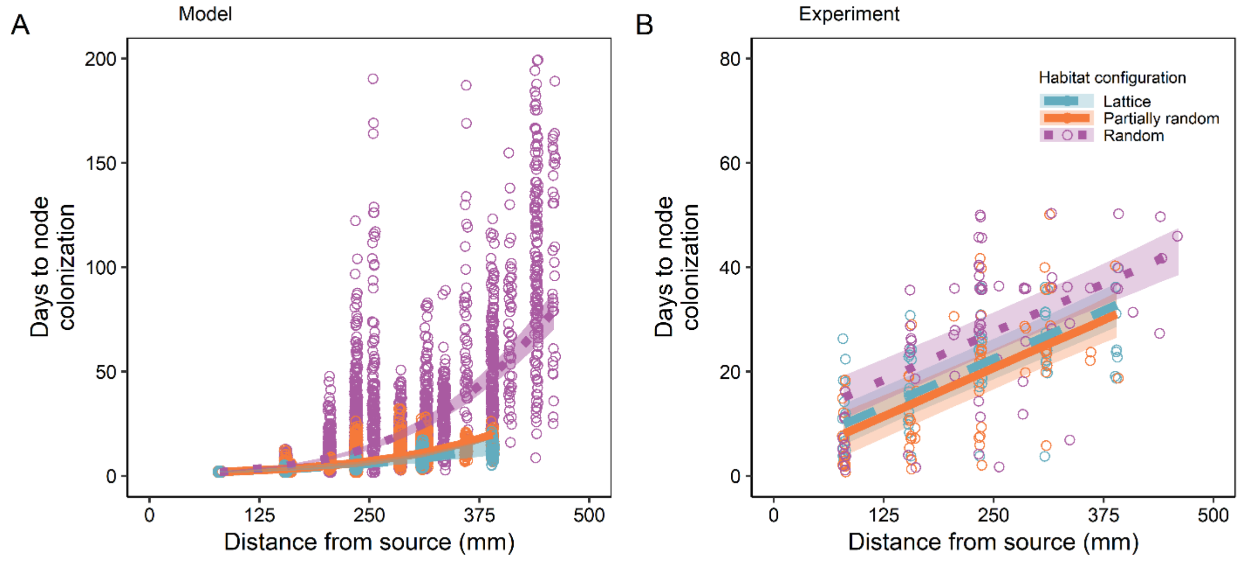

Figure S10. Habitat configuration affects time to node colonization after accounting for minimum distance from source. Left panel shows model predictions for *F. candida*, assuming the exponent of the dispersal kernel, *b* = -0.0430; right panel shows experiment results. Regression lines and bands show predicted values from linear mixed models with 95% confidence intervals. Points represent single nodes. In both the model and experiment, the effect of habitat configuration remains after controlling for the distance between the source and focal node (LMM for experimental data: $\chi^2 = 6.61$, df = 2, p = 0.037). *Sample sizes:* each model treatment is represented by 50 replicate simulations; each experiment treatment is represented by 8 replicate networks.

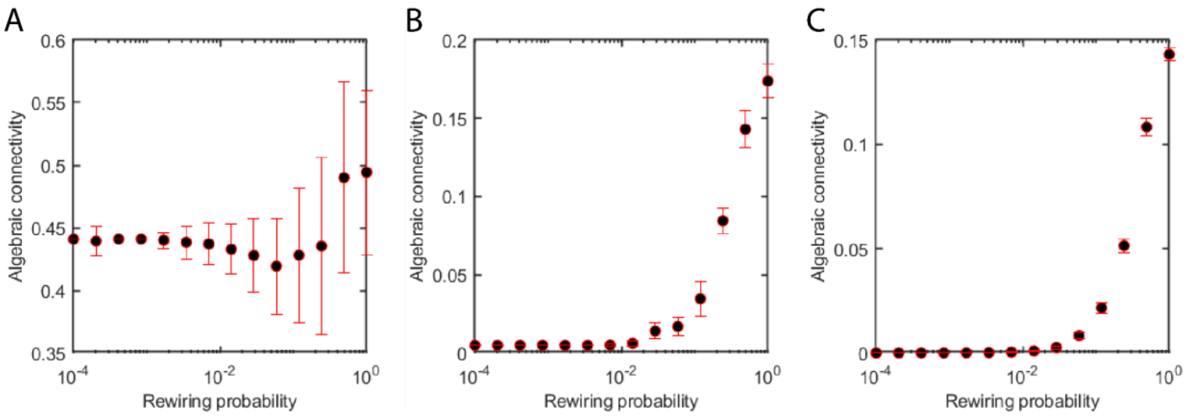

Figure S11. Algebraic connectivity as a function of rewiring probability for networks of three different sizes. *A*) 10 nodes, *B*) 100 nodes, *C*) 1000 nodes. When the rewiring probability is zero the network is a lattice, and when the rewiring probability is 1 the network is an Erdös-Rényi random network. Rewiring is performed following (1). All networks have an average degree of 4. Algebraic connectivity is calculated over the normalized Laplacian matrix of the binary network.

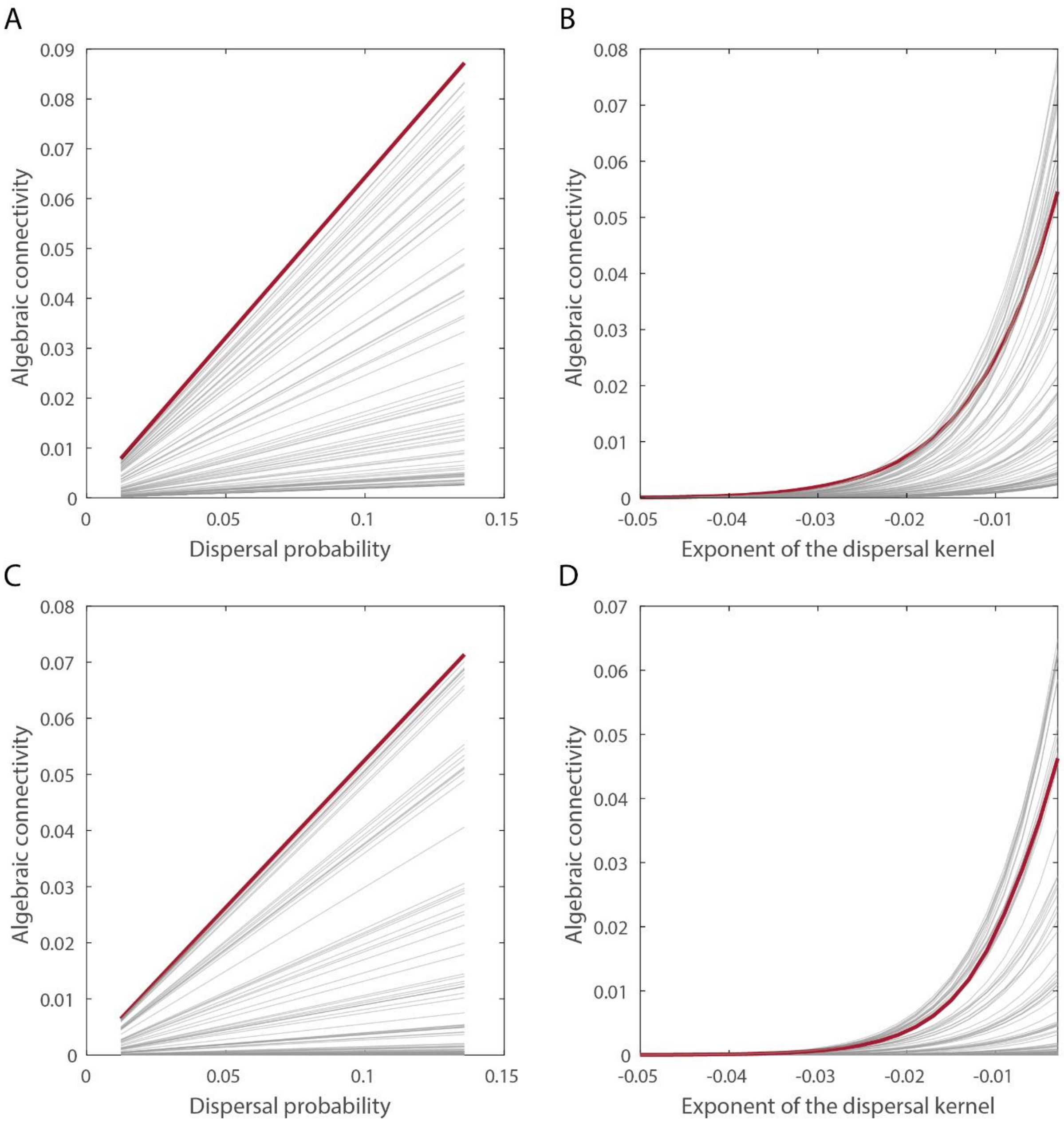

Figure S12. Algebraic connectivity in networks whose weights represent the dispersal probability between two habitat fragments. Each line represents one network. *A*) The dispersal probability is the same in all links, regardless of the distance between two nodes. This scenario would be equivalent of models that ignore distance or the geographical embedding of the spatial networks. *B*) The dispersal probability decreases as a function of the distance between the habitat fragments following a negative exponential. In order to make the left panels and right panels comparable, the dispersal probability in *A* and *C* is calculated as the average dispersal probability for a given dispersal kernel in *B* and *D*. In *A* and *B* we used networks of 100 nodes while *C* and *D* are equivalent to *A* and *B* but with networks of 1000 nodes.

Table S2. Comparison between algebraic connectivity (AC) and network diameter (ND) at explaining days to full network occupancy for the experimental data. Network diameter is the shortest distance between the two most distant nodes in the network. That distance would be given in number of links for the binary networks and in mm for the weighted networks. Comparisons are meant to be drawn between AC and ND and not across Weighted and Binary.

	**Network**	**Metric**	**Estimate**	**Std. Error**	**F. Value**	**DF**	**p-value**	**R^2**
Experiment	Binary	AC	11.1871	5.1427	4.7	1.22	0.0406	0.14
Experiment	Binary	ND	-0.0840	0.0565	2.2	1.22	0.1508	0.05
Experiment	Weighted	AC	-3.9073	1.1095	12.4	1.22	0.0019	0.33
Experiment	Weighted	ND	0.0007	0.0002	11.3	1.22	0.0029	0.31

Table S3. Comparison between algebraic connectivity (AC) and network diameter (ND) at explaining days to full network occupancy for the model data when using the dispersal kernel parameterized for *F. candida*. Network diameter is the shortest distance between the two most distant nodes in the network. That distance would be given in number of links for the binary networks and in mm for the weighted networks. Comparisons are meant to be drawn between AC and ND and not across Weighted and Binary.

	**Network**	**Metric**	**Spearman's rho**
Model	Binary	AC	0.40
Model	Binary	ND	-0.35
Model	Weighted	AC	-0.78
Model	Weighted	ND	0.80

SI References

1. D. J. Watts, S. H. Strogatz. Collective dynamics of 'small-world' networks. *Nature*. 393, 440-442 (1998).
2. M. T. Fountain, S. P. Hopkin. *Folsomia candida* (Collembola): a "standard" soil arthropod. *Annu Rev Entomol*. 50, 201-222 (2005).
3. S. P. Hopkin. Biology of the springtails (Insecta: Collembola) (Oxford University Press, Oxford, 1997).
4. L. J. Gilarranz, B. Rayfield, G. Liñán-Cembrano, J. Bascompte, A. Gonzalez. Effects of network modularity on the spread of perturbation impact of experimental metapopulations. *Science*. 357, 199-201 (2017).
5. J. R. Beddington. Age distribution and the stability of simple discrete time population models. *J. Theor. Biol*. 47, 65-74 (1974).
6. A. Gonzalez, R. D. Holt. The inflationary effects of environmental fluctuations in source-sink systems. *Proc Natl Acad Sci USA*. 99, 14872-14877 (2002).
7. M. Fiedler. Algebraic connectivity of graphs. *Czech. Math. J*. 23, 298-305 (1973).
8. R Core Team. R: A language and environment for statistical computing (R Foundation for Statistical Computing, Vienna, Austria, 2020).
9. D. Bates, M. Maechler, B. Bolker, S. Walker. Fitting linear mixed-effects models using lme4. *J. Stat. Soft*. 67, 1-48 (2015).
10. A. Canty, B. Ripley boot: Bootstrap R (S-Plus) functions. (2020).
11. A. C. Davison, D. V. Hinkley. Bootstrap methods and their applications (Cambridge University Press, Cambridge, 1997).
